# Comparison of zebrafish and mice knockouts for Megalencephalic Leukoencephalopathy proteins indicates that GlialCAM/MLC1 forms a functional unit

**DOI:** 10.1186/s13023-019-1248-5

**Published:** 2019-11-21

**Authors:** Carla Pérez-Rius, Mónica Folgueira, Xabier Elorza-Vidal, A. Alia, Maja B. Hoegg-Beiler, Muhamed N. H. Eeza, María Luz Díaz, Virginia Nunes, Alejandro Barrallo-Gimeno, Raúl Estévez

**Affiliations:** 1 Unitat de Fisiologia, Departament de Ciències Fisiològiques, Genes Disease and Therapy Program IDIBELL-Institute of Neurosciences, Universitat de Barcelona, L’Hospitalet de Llobregat, Barcelona, Spain; 2 Department of Biology, Faculty of Sciences, University of A Coruña, 15008-A Coruña, Spain; 3 Centro de Investigaciones Cientificas Avanzadas (CICA), University of A Coruña, 15008-A Coruña, Spain; 4 Leiden Institute of Chemistry, Leiden University, Leiden, The Netherlands; 5 Institute of Medical Physics and Biophysics, University of Leipzig, Leipzig, Germany; 6 Leibniz-Forschungsinstitut für Molekulare Pharmakologie (FMP), Department Physiology and Pathology of Ion Transport, D-13125 Berlin, Germany; 7 Max-Delbruck-Centrum für Molekulare Medizin (MDC), D-13125 Berlin, Germany; 8 Centro de Investigación en red de enfermedades raras (CIBERER), ISCIII, Madrid, Spain; 9 Unitat de Genètica, Departament de Ciències Fisiològiques, Genes Disease and Therapy Program IDIBELL, Universitat de Barcelona, L’Hospitalet de Llobregat, Barcelona, Spain; 10 Facultat de Medicina, Departament de Ciències Fisiològiques, Universitat de Barcelona-IDIBELL, C/Feixa Llarga s/n 08907 L’Hospitalet de Llobregat, Barcelona, Spain

**Keywords:** *MLC1*, *GLIALCAM*, Megalencephalic leukoencephalopathy, Myelin, Astrocyte, Zebrafish

## Abstract

**Background:**

Megalencephalic Leukoencephalopathy with subcortical Cysts (MLC) is a rare type of leukodystrophy characterized by astrocyte and myelin vacuolization, epilepsy and early-onset macrocephaly. MLC is caused by mutations in *MLC1* or *GLIALCAM*, coding for two membrane proteins with an unknown function that form a complex specifically expressed in astrocytes at cell-cell junctions. Recent studies in *Mlc1*^−/−^ or *Glialcam*^−/−^ mice and *mlc1*^*−/−*^ zebrafish have shown that MLC1 regulates glial surface levels of GlialCAM in vivo and that GlialCAM is also required for MLC1 expression and localization at cell-cell junctions.

**Methods:**

We have generated and analysed *glialcama*^*−/−*^ zebrafish. We also generated zebrafish *glialcama*^−/−^ *mlc1*^*−/−*^ and mice double KO for both genes and performed magnetic resonance imaging, histological studies and biochemical analyses.

**Results:**

glialcama^−/−^ shows megalencephaly and increased fluid accumulation. In both zebrafish and mice, this phenotype is not aggravated by additional elimination of *mlc1*. Unlike mice, mlc1 protein expression and localization are unaltered in *glialcama*^−/−^ zebrafish, possibly because there is an up-regulation of *mlc1* mRNA. In line with these results, MLC1 overexpressed in *Glialcam*^−/−^ mouse primary astrocytes is located at cell-cell junctions.

**Conclusions:**

This work indicates that the two proteins involved in the pathogenesis of MLC, GlialCAM and MLC1, form a functional unit, and thus, that loss-of-function mutations in these genes cause leukodystrophy through a common pathway.

## Background

One of the most important functions that astrocytes perform is buffering the increase in potassium that occurs during neuronal firing to help restore baseline conditions [1]. Astrocytes buffer excess potassium through different pathways in a still undefined manner: mainly via the Na^+^, K^+^, ATPase pump, but also using the Na^+^, K^+^, Cl^−^ co-transporter, the potassium channel Kir4.1 and through gap-junction dependent processes [2]. It has also been suggested that the ClC-2 chloride channel may play a role in glial potassium accumulation [3, 4]. Animal models deficient in proteins involved in this process (Kir4.1, ClC-2, Cx32/Cx47, Cx30/Cx43) show several defects in potassium clearance, increased neuronal excitability and presence of vacuoles in myelin [5–8]. Since water movement is parallel to ion flow, it is possible that vacuoles are a consequence of an impaired ion uptake. Additionally, potassium and water entry into astrocytes also causes cellular swelling. A swelling-dependent chloride channel named VRAC (for Volume-Regulated Anion Channel) strongly expressed in astrocytes is then activated, releasing chloride and osmolytes from the cell, thus changing the driving force for water movement and restoring the astrocyte’s original size [9].

A similar phenotype to what is present in knockout animals of genes involved in potassium clearance [5–8] has been observed in patients affected with Megalencephalic Leukoencephalopathy with subcortical Cysts (MLC), a rare type of leukodystrophy [10]. MLC is characterized by astrocyte and myelin vacuolization, epilepsy and early-onset macrocephaly [11]. The epilepsy and the presence of vacuoles in MLC patients suggested a possible defect in potassium handling [10]. MLC is caused by mutations in either *MLC1* [12] or *GLIALCAM* [13]. *MLC1* encodes for a membrane protein with eight predicted transmembrane domains (MLC1), which is specifically expressed in astrocytes at cell-cell junctions, including the Bergmann glia of the cerebellum and highly enriched in their perivascular endfeet contacting the blood brain barrier (BBB) [14, 15]. GlialCAM is an adhesion molecule of the immunoglobulin superfamily expressed predominantly in astrocytes and oligodendrocytes [15, 16].

The pathophysiological mechanisms leading to MLC are unclear [17]. Apart from the phenotype of MLC patients, some experimental evidence suggest that GlialCAM/MLC1 have a role in potassium clearance: i) GlialCAM is an auxiliary subunit of the ClC-2 chloride channel [18]. GlialCAM makes ClC-2 an ohmic channel due to a change in its gating mechanism [19], which allow mediating chloride influx at depolarized potentials [15], as expected for a chloride channel involved in potassium uptake; ii) in astrocyte cultures, localization of GlialCAM, MLC1 and ClC-2 at cell-cell junctions depend on extracellular potassium [20]; iii) mice models deficient for *Mlc1* or *Glialcam* display altered brain potassium dynamics [21] and iv) astrocytes deficient in MLC1 or GlialCAM show reduced VRAC activity [22–24]. Even though this experimental evidence suggested the involvement of MLC1 and GlialCAM proteins in potassium uptake, the molecular basis of these defects is unclear, as the precise functions of MLC1 of GlialCAM are still unknown.

The biochemical relationships between MLC1 and GlialCAM are also not well defined. In cultured cell lines such as HeLa cells, MLC1 cannot reach cell junctions without GlialCAM, whereas GlialCAM expressed alone is located at cell-cell junctions [25]. In agreement with this in vitro data, mice deficient in *Glialcam* show a mislocalization of Mlc1 [15, 16]. On the other hand, MLC1 expressed alone in cell lines can reach the plasma membrane [26–28], while in *Glialcam* knockout mice, Mlc1 is not present at the plasma membrane and Mlc1 protein levels are reduced [15, 16]. Considering that in primary astrocytes, GlialCAM improves the plasma membrane localization of MLC-related mutants of MLC1 that present folding defects, it has been suggested that GlialCAM has two putative roles: bringing MLC1 at cell-cell junctions and stabilizing MLC1 [22].

Unexpectedly, both mice [14, 15] and zebrafish [29] deficient in MLC1 also show a mislocalization of GlialCAM in astrocytes and oligodendrocytes. However, this mislocalization is observed in Bergmann glia [29] but not in astrocytes surrounding blood vessels [25] in humans. Furthermore, in astrocyte cultures from *Mlc1*^−/−^ mice, GlialCAM is not mislocalized, but it loses its localization at cell-cell junctions after incubating astrocytes with a depolarizing solution [29]. According to this, it has been suggested that the mislocalization of GlialCAM when MLC1 is not present depends on the extracellular potassium concentration by an undefined mechanism involving signal transduction processes [20, 23, 30, 31].

In summary, although MLC1 and GlialCAM proteins form a complex located at cell-cell junctions, the biochemical role of each protein in this complex is not well defined. In the present work, with the aim of understanding this relationship, we have generated and analyzed zebrafish deficient in *glialcama* as well as zebrafish and mice deficient in both proteins. Two orthologous genes for GlialCAM have been described in zebrafish (*glialcama* and *glialcamb*), although previous results suggested that glialcama is the orthologous gene of *GLIALCAM* [29]. The characterization of these models has provided new insights into the molecular basis of GlialCAM and MLC1 interactions.

## Methods

### Zebrafish maintenance

Zebrafish were kept at the animal facility in Bellvitge Campus, University of Barcelona, under standard conditions at 28 °C, 14 h/10 h light/dark period. AB or AB/TL strains were used in all the experiments. All experimental procedures conformed to the European Community Guidelines on Animal Care and Experimentation and were approved by animal care and use committees.

### Generation of *glialcama* knockout zebrafish

We designed a pair of TALE nucleases to target two sequences at the beginning of glialcama exon1: CTGCTCTCAAGATGAAGGCA (where the start codon is underlined) and TGAAGGAATGGCTGTCTCT, leaving a 20 bp spacer: GAGCGGGAGGCATCATGCAA (BsrBI restriction site underlined). Plasmids containing the TALE nucleases were synthesized by GeneArt (then Life Technologies), and then cloned by Gateway into pCS2-destination vector. Plasmids were linearized with KpnI and mRNAs were synthesized with mMessage mMachine (Ambion). One hundred pg of each TALE Nuclease mRNA were injected into one cell zebrafish embryos, DNA was isolated from pooled embryos at 3dpf and the target sequence amplified with the following primers: GCCCTGAGTGGACAAATCAT and AAACTGACAACAGCGCACAC to check if the BsrBI restriction site was lost due to the action of the TALE nucleases and the subsequent mistakes made by the cellular repair mechanisms. The remaining embryos were raised to adulthood and crossed with wild-type animals. The heterozygosity of their offspring was confirmed by PCR and High Resolution melting Analysis (HRMA) on a StepOne PCR machine (Invitrogen). These F1 embryos were raised to adulthood, tail clipped and genotyped. PCR products were cloned by TA cloning into the pGEMt vector (Promega). Individual colonies were sequenced using T7 and SP6 primers to characterize the mutations generated.

### Molecular biology

Plasmids used were constructed using standard molecular biology techniques employing recombinant PCR and the multisite gateway system (Life Technologies). The integrity of all cloned constructs was confirmed by DNA sequencing.

### RT-PCR

Adult zebrafish were euthanized using an overdose of tricaine (MS222, Sigma). Adult tissues were quickly dissected and flash-frozen in liquid nitrogen. Total RNA was isolated with TRIzol and retrotranscribed using random hexamers with the SuperScript IV system (Life Technologies). The oligonucleotides pairs used for qPCR are the following: *Rpl13a* (internal control), sense: TCTGGAGGACTGTAAGAGGTATGC, anti-sense: TCTGGAGGACTGTAAGAGGTATGC; *mlc1*, sense: GCACGTTCAGTGGACAACTG, anti-sense: CACAATCATTGGGCCTTCAG; *glialcama*, sense: CCCACCCACCAAGACTAAGC, anti-sense: CATCCTCAGTCGTGCTCATCTG; *glialcamb*, sense: AGACCGGATCTTGGTGTTTGA, anti-sense: TAGGCTCATCCACAGTGAGATTGA.

qPCR was performed with SYBR Select reagent (Life Technologies) in a StepOne apparatus (Life Technologies). Three experiments were analyzed, with three replicate samples in each experiment. The expression levels were normalized using the comparative Ct method normalized to the internal control genes. The final results were expressed as the relative messenger RNA (mRNA) levels as indicated in the corresponding figures, taking into account the efficiency of each primer with the Pfaffl method.

### Histological staining methods in zebrafish

Fish were deeply anesthetized in 0.1% tricaine methanosulfonate (Sigma, MS-222) in fresh water and fixed by vascular perfusion with 4% paraformaldehyde in 0.1 M phosphate buffer (PB). Fish heads were post-fixed in the same fixative for at least 24 h at room temperature. Next, brains and eyes were extracted, cryopreserved in 30% sucrose in PB, frozen with liquid-nitrogen-cooled methylbutane and cut in a cryostat. Transverse sections (12–14 μm thick) were collected onto gelatinized slides.

For immunohistochemistry, sections were rinsed in saline phosphate buffer (PBS) and sequentially incubated at room temperature with: (1) normal goat serum (NGS, Sigma, 1:10 in PBS) for 1 h; (2) primary antibody or cocktail of primary antibodies, overnight (for antibodies and dilutions, see below); (3) PBS for 15 min; (4) secondary fluorescent antibody or cocktail of fluorescent antibodies for 1 h (for antibodies and dilutions, see below); (6) PBS for 15 min. Incubations with primary and secondary antibodies were made at room temperature in a humid chamber. Finally, sections were mounted using 50% glycerol in PB.

Primary antibodies and dilutions used in the study were: rabbit anti-zebrafish mlc1 (1:100) and rabbit anti-zebrafish glialcama (1:100). The secondary antibody used was goat anti rabbit- Alexa Fluor 488 (Invitrogen, 1:500). All dilutions were done in 10% NGS in PBS. Negative controls omitting incubation with primary antibody were performed, showing no unspecific immunoreactivity.

Sections were first observed in a Nikon Eclipse Fluoresencent microscope and then selected sections of were imaged in a Nikon A1R confocal microscope. Confocal and fluorescent data was processed and analysed using ImageJ software.

### MRI imaging in zebrafish

Magnetic resonance microimaging (μMRI) of Zebrafish was performed on a vertical wide-bore 7 T Bruker Avance 300WB spectrometer, with a 1000 mT·m^− 1^ actively shielded imaging gradient insert (Bruker Biospin GmbH, Germany). The system was interfaced to a Linux PC running Topspin 2.0 and ParaVision 3.2 software (Bruker Biospin GmbH, Germany). For RF excitation and detection, a birdcage radio-frequency (RF) coil with an inner diameter 10 mm was used. For μMRI, adult zebrafish were euthanized and fixed in 4% buffered paraformaldehyde (Zinc Formal-Fixx, ThermoShandon, UK) for 7 days and subsequently embedded in Fomblin (Solvay Solexis, Inc.) to avoid any artefacts that may arise due to magnetic susceptibility differences at air–tissue boundaries. The magnetic field homogeneity was optimized by shimming before each μMRI measurement. For position determination and selection of the desired region, each session of measurements began with a multislice orthogonal gradient-echo sequence. Subsequently, high resolution *T*_*2*_ weighted images were acquired by using a rapid acquisition with relaxation enhancement (RARE) sequences with repetition time (TR) = 3000 ms; effective echo time (TE) = 18 ms; RARE factor = 4; slice thickness 0.2 mm; field of view 1.2 × 1.2 mm; image matrix of 256 × 256 pixels, resulting in a spatial resolution of 47 μm.

For transverse relaxation time (*T*_*2*_*)* measurement, a standard multi-slice multi-echo (MSME) sequence was used. This sequence is based on the Carr-Purcell Meiboom-Gill (CPMG) sequence, where transverse magnetization of a 90° pulse is refocused by a train of 180° pulses generating a series of echoes. The following imaging parameters were used: nominal flip angles = 90° and 180°, and a train of 12 echoes with TEs ranging from 8.17 ms to 98 ms with 8.17 ms echo-spacing; TR = 2 s, slice thickness 0.5 mm; number of slices 8 and a matrix size 256 × 256 pixels.

For calculation of *T*_*2*_ relaxation time, regions of interest (ROIs) were drawn at various locations within the zebrafish brain using an image sequence analysis (ISA) tool package (Paravision 5, Bruker). Another ROI in the muscle was used as an internal control. Monoexponential fitting was then used to calculate *T*_*2*_ using a monoexponential fit function [y = A+ C*exp. (−t/*T*_*2*_)], where A = Absolute bias, C = signal intensity, *T*_*2*_ = transverse relaxation time. Means and standard deviation for *T*_*2*_ relaxation times for each ROI were calculated.

For measurement of brain areas, the desired telencephalone and whole brain regions were drawn on the image and areas were computed using an image sequence analysis (ISA) tool package (Paravision 5, Bruker). The data were exported to OriginPro v. 8 (OriginLab, Northampton, MA, USA) for further analysis and percentage of Telencephalon with respect to whole brain area was calculated. One-way ANOVA (Bonferroni’s post-test) for comparison of mean between each group was performed. Levene’s test was performed for homogeneity of variance analysis.

### Mouse studies

The generation of *Glialcam*^−/−^ and *Mlc1*^−/−^ mice has been previously described [15]. For histological analyses of brains, mice were perfused with 4% PFA/PBS and organs were postfixed overnight. Haematoxylin–eosin staining was performed on 6 μm paraffin sections of brains.

Mouse primary astrocyte cultures were prepared from cortex and hippocampus, which were removed from newborn mice. Astrocyte cultures were prepared from 0 to 1 day old OF1 mice. Cerebral cortices were dissected and the meninges were carefully removed in cold sterile 0.3% BSA, 0.6% glucose in PBS. The tissue was trypsinized for 10 min at 37 °C and mechanically dissociated through a small bore fire-polished Pasteur pipette in complete DMEM medium (Dulbecco’s Modified Eagle’s Medium with 10% heat-inactivated fetal bovine serum (Biological Industries), 1% penicillin/streptomycin (Invitrogen) and 1% glutamine (Invitrogen) plus 40 U/ml DNase I (Sigma)). The cell suspension was pelleted and re-suspended in fresh complete DMEM, filtered through a 100-μm nylon membrane (BD Falcon) and plated into 75 cm^2^ cell culture flasks (TPP). When the mixed glial cells reached confluence, contaminating microglia, oligodendrocytes and precursor cells were dislodged by mechanical agitation and removed. Astrocytes were plated in 6-well plates, at density of 4·10^5^ cells per well, or in poly-D-lysine-coated cover slips at 7.5·10^4^cells in 24-well plates. Medium was changed every 3 days. In order to obtain astrocyte cultures arrested in the cell cycle, medium was replaced and cytosine β-D-arabinofuranoside (AraC, Sigma) (2 μM) was added. Cultured astrocytes were identified by their positive GFAP (Glial Fibrillary acid protein) staining (Dako), being > 95% of cells GFAP positive.

For Western blot studies, astrocyte lysates were prepared by homogenization of cells in PBS containing 1% Triton X-100 and protease inhibitors: 1 μM Pepstatin and Leupeptin, 1 mM Aprotinin and PMSF, incubated for 1 h at 4 °C and centrifugated. Supernatants were quantified using BCA kit (Pierce) and mixed with SDS loading sample buffer. After SDS PAGE, membranes were incubated with primary antibodies: anti-MLC1 (1:100), anti-GlialCAM (1:100) and anti-β-Actin (1:10000, Sigma) and secondary antibodies: HRP-conjugated anti-rabbit and anti-mouse (1:10000; Jackson). Quantification of Western blots was performed by ImageJ at different exposition times to ensure linearity.

## Results

### Generation and characterization of zebrafish *glialcama* knockout

We previously described that the teleost-specific genome duplication yielded two *glialcam* paralogues: *glialcama* and *glialcamb* [29]. Experimental evidence suggests that glialcama and not glialcamb exerts similar functions to its orthologue GlialCAM: i) when expressed transiently in cell lines, glialcama is detected in cell junctions, while glialcamb is intracellular [29]; ii) glialcama is able to target MLC1 and ClC-2 to cell junctions in cell lines, but not glialcamb [29]; iii) glialcama modifies the functional properties of human and zebrafish ClC-2 proteins expressed in *Xenopus* oocytes, whereas glialcamb reduces ClC-2 function [32]; iv) it has been shown that in *mlc1*^−/−^ glialcama is mislocalized [29], as happens with GlialCAM in *Mlc1*^−/−^ mice [15] and v) mlc1 and glialcama could be co-immunoprecipitated (Additional file 1: Figure S1).

**Fig. 1:**
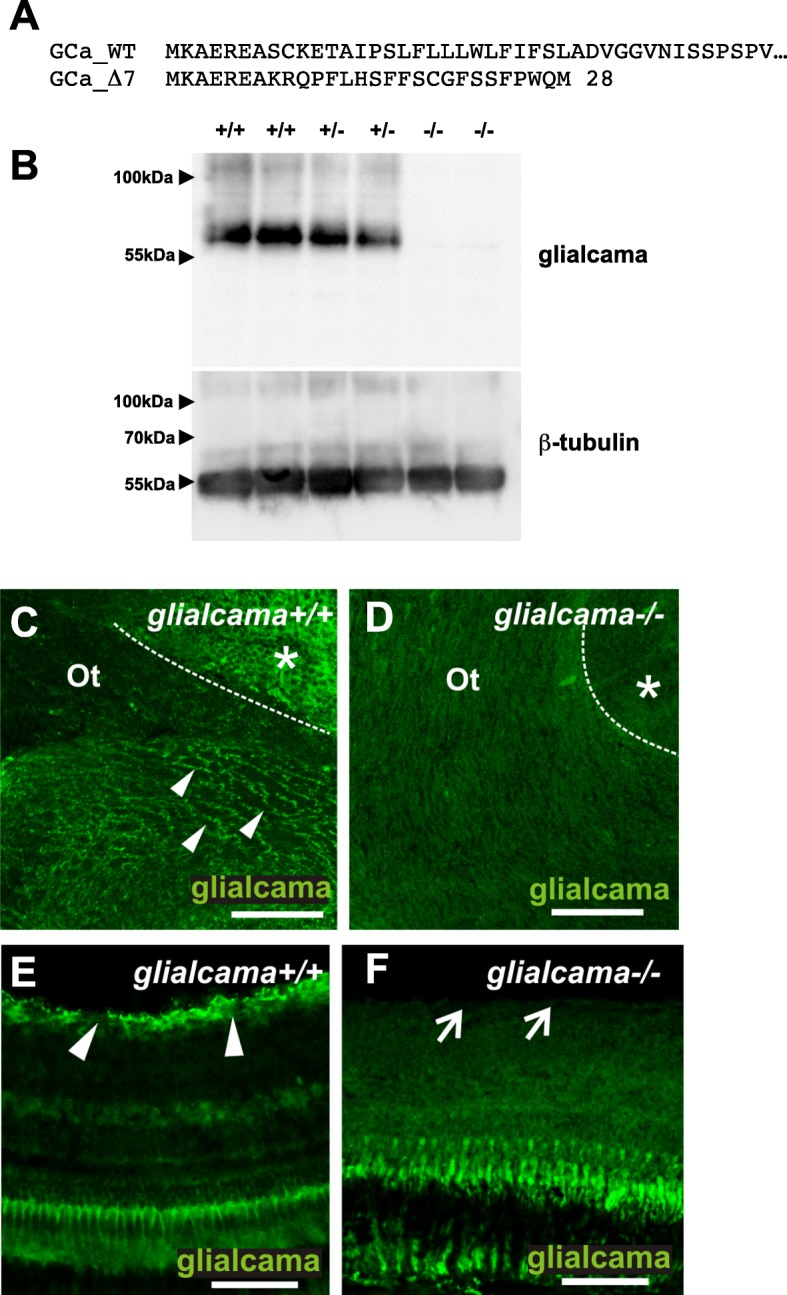
Generation of a *glialcama* knockout line in zebrafish. **a** An allele with a deletion of 7 nucleotides in *glialcama* (Δ7, from now on −/−) was generated using a TALEN nuclease. The deletion generates an early stop codon, resulting in a protein of only 28 amino acids in lenght. **b** Western blot of brain extracts from adult wild-type (+/+), heterozygous (+/−) or homozygous mutant (−/−) fish for the *glialcama* knockout allele demonstrates the absence of the glialcama protein in the homozygous zebrafish. **c** glialcama expression in the optic tract (Ot) (arrowheads) and diffusely in cell bodies of the preoptic region (asterisk) of wild type fish (dotted line: optic tract/preoptic region boundary). **d** No glialcama immunoreactivity is observed in the optic tract or brain parenchyma of *glialcama* knockout fish. Dotted line: optic tract/preoptic region boundary. Asterisk: preoptic region. **e** glialcama immunoreactivity is observed in the inner limiting membrane of the wild type retina (arrowheads). **f** No glialcama immunoreactivity is observed in knockout retina. Expression observed in *glialcama*^−/−^ represents autofluorescence in photoreceptors that was consistently observed, even in the case of control immunostainings with secondary antibody only. Arrows point to the inner limiting membrane. Scale bars: 60 μm (**e**, **f**); 75 μm (**c**, **d**)

Using TALEN nucleases (see Methods), we generated a zebrafish *glialcama* knockout line that carries a deletion of 7 nucleotides (Δ7) in the first exon of the *glialcama* gene. The deletion changes the open reading frame after the seventh amino acid and causes a premature stop codon at amino acid 28 (Fig. 1a). To verify that this mutation abolished the glialcama protein, we assayed its expression in brain extracts from wild-type, heterozygous and homozygous *glialcamaΔ7* adult fish siblings (Fig. 1b). No glialcama protein expression could be detected in homozygotes, validating *glialcamaΔ7* as a *glialcama* knockout line (*glialcama*^bcn1^), which we will refer to as *glialcama*^−/−^ zebrafish from now on. As with the *Glialcam*^−/−^ mouse or the *mlc1*^−/−^ zebrafish [15, 29], the homozygous *glialcama*^−/−^ zebrafish turned out to be viable and fertile, with the expected mendelian ratio among adult descendants. Previous immunofluorescence experiments detected similar localization of glialcama and mlc1 in radial glial cell bodies and their processes in the brain (Fig. 1c and [29]) and in the retina, where they are highly expressed at Müller glia end-feet at the inner limiting membrane (Fig. 1e and [29]). We verified that the previously observed glialcama localization was specific, as immunofluorescence studies confirmed no expression in the *glialcama*^−/−^ fish neither in the brain nor in the retina (Fig. 1d and f, respectively).

### Comparison of mouse and zebrafish MLC knockout phenotypes

Histopathology of brain sections from *Mlc1*^−/−^ and *Glialcam*^−/−^ mice revealed the presence of vacuolization mainly in fibre tracts of the cerebellum [15, 16, 21, 33]. In addition, measurements of brain volume revealed that the whole brain is bigger in MLC knockout models than in its wild-type littermates [14, 16, 29]. No major differences were found in the vacuolization phenotype between both mice models [15]. Regarding the zebrafish models, in *mlc1*^−/−^ animals, MRI showed that the telencephalon is larger in comparison to the wild-type, and there are several lesions due to increased fluid in the telencephalon and mesencephalon [29].

**Fig. 2:**
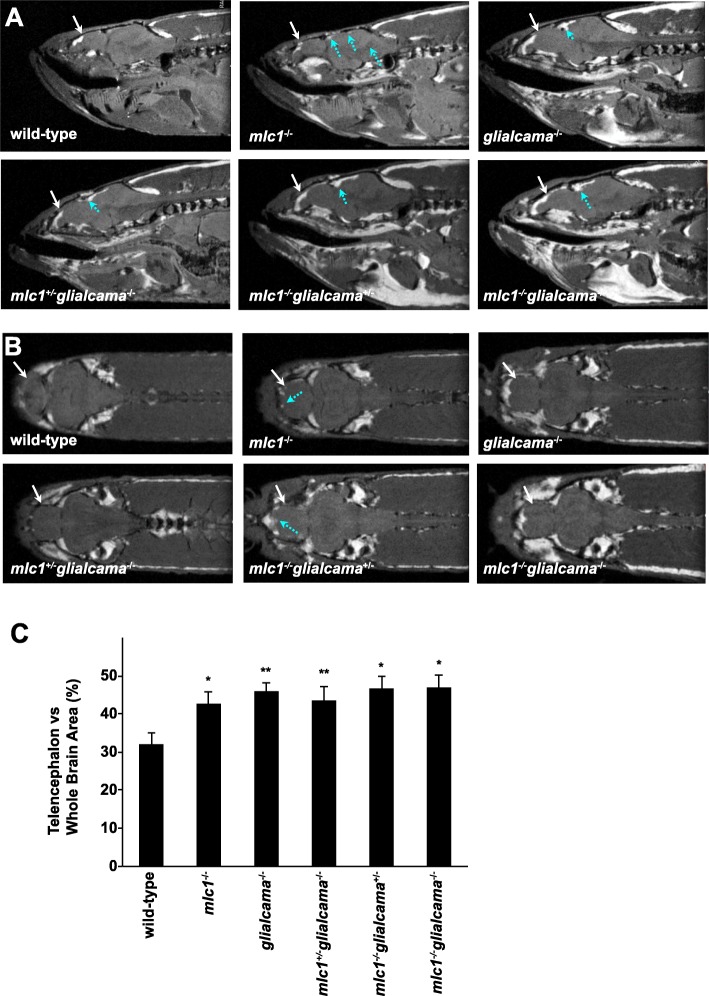
MR images of wild-type and various zebrafish mutants. **a** The sagittal slices were obtained with an in-plane resolution of 47 μm. The size of telencephalon was bigger in mutant zebrafish as compared to wild type (white arrows). Lesions in mesencephalon in mutants are shown with blue arrows. **b** The coronal slices were obtained with an in-plane resolution of 47 μm. The size of telencephalon was bigger in mutant zebrafish as compared to wild type (white arrows). Lesions in telencephalon are shown with blue arrows. **c** Telencephalon vs. whole brain area (%). In order to compare the size of the telencephalon relative to the whole brain of the different groups one-way analysis of variance (ANOVA) was performed and indicated that the size was larger in all mutants as compared to wild type (*p < 0.05; **p < 0.005) (*n* = 3). No statistical differences were observed in the percent area of telencephalon versus whole brain size between single knockout zebrafish for one gene with the single knockout/heterozygous or the double knockout (*p* > 0.05) (see Additional file 3: **Table S1**)

Therefore, we analysed the brain phenotype of *glialcama*^−/−^ zebrafish by MRI. Furthermore, *glialcama*^−/−^ and *mlc1*^−/−^ zebrafish were pair-wise mated to obtain animals knockout for both genes or knockout for a single gene and heterozygous for the other. Sagittal (Fig. 2a) and coronal (Fig. 2b) MR images of wild-type and various zebrafish mutants were obtained and analysed. We observed similar lesions in *glialcama*^−/−^ zebrafish to what has been previously observed for the *mlc1*^−/−^ zebrafish [29]. Analysis of T_2_ relaxation time in the healthy and damaged brain regions showed similar values for lesions and the ventricles, indicating that lesions were due to increased fluid (Additional file 2: Figure S2). Furthermore, as in *mlc1*^−/−^ animals [29], the size of the telencephalon relative to the whole brain was also larger in the *glialcama*^−/−^ (Fig. 2c and Additional file 3: Table S1). These results indicate that the lack of glialcama causes two typical MLC features: megalencephaly and increased fluid accumulation. Furthermore, they also suggest that glialcamb does not compensate for the lack of glialcama. For this reason, we did not analyze whether glialcamb could be co-immunoprecipitated with mlc1 and we did not generate *glialcamb*^−/−^ fish.

We further compared the phenotype of the single knockout zebrafish for one gene with the single knockout/heterozygous or the double knockout. No statistical differences were observed in the percent area of telencephalon after normalizing versus whole brain size neither in the amount of damaged brain regions (Fig. 2c and Additional file 3: Table S1).

**Fig. 3:**
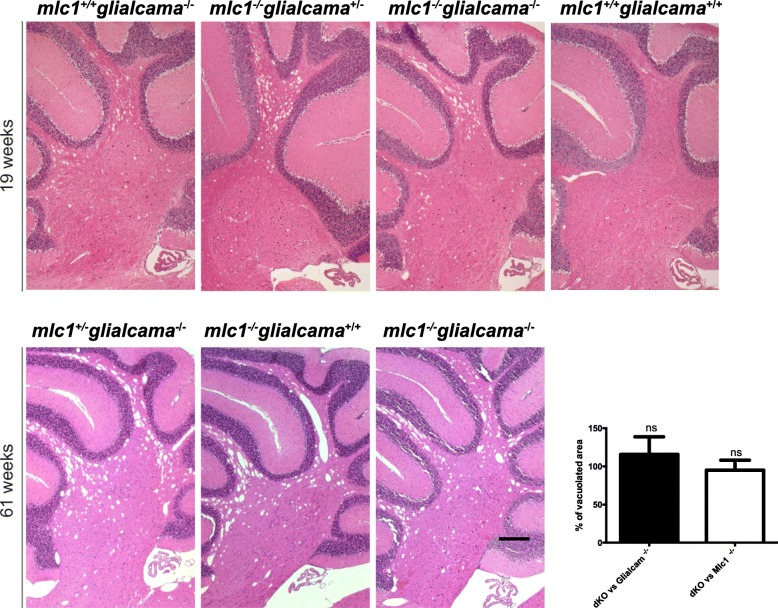
Myelin vacuolization in *Glialcam*^−/−^, *Mlc1*^−/−^ and *Glialcam*^−/−^*Mlc1*^−/−^ mouse models. Haematoxylin-eosin staining of sagittal sections of the cerebellum of 19- and 61-week-old mice showed similar levels of myelin vacuolization in *Glialcam*^−/−^, *Mlc1*^−/−^ and *Glialcam*^−/−^*Mlc1*^−/−^ animals. As a control we show the same area of a wild-type mouse at 19 weeks. The inset shows the percentage of vacuolation in double KO animal versus the vacuolation observed in *Glialcam* KO animals (*n* = 3) and *Mlc1* KO animals (*n* = 3) considering each age independently, without substracting the minor vacuolization observed in wild-type animals. Data were analyzed by GraphPad Prism software. In order to compare the different groups (dKO vs *Glialcam*^−/−^ and dKO vs *Mlc1*^−/−^), one-way analysis of variance (ANOVA) followed by post-hoc Bonferroni’s multiple comparison test was used. ns: not significative. Scale bar, 400 μm

To study if this was also the case in mice, we analysed the extent of myelin vacuolization in fibre tracts of the cerebellum in single or double knockout mice for *Mlc1* and *Glialcam* (Fig. 3). Additional loss of *Glialcam* in *Mlc1*^−/−^/*Glialcam*^−/−^ mice did not increase the degree of vacuolization over that observed for *Mlc1*^−/−^ or *Glialcam*^*−/−*^ mice. As previous studies on double knockout mice for both *Clcn2* and *Glialcam* revealed that incremental effects on vacuolation are readily observed [15], we conclude that no such incremental effects occurred in *Glialcam* / *Mlc1* double knockout mice.

Thus, in both animal models (mice and zebrafish) deletion of both genes simultaneously did not exacerbate the brain phenotype of the single knockouts.

### Expression and localization of mlc1 is unaltered in zebrafish *glialcama*^−/−^

**Fig. 4:**
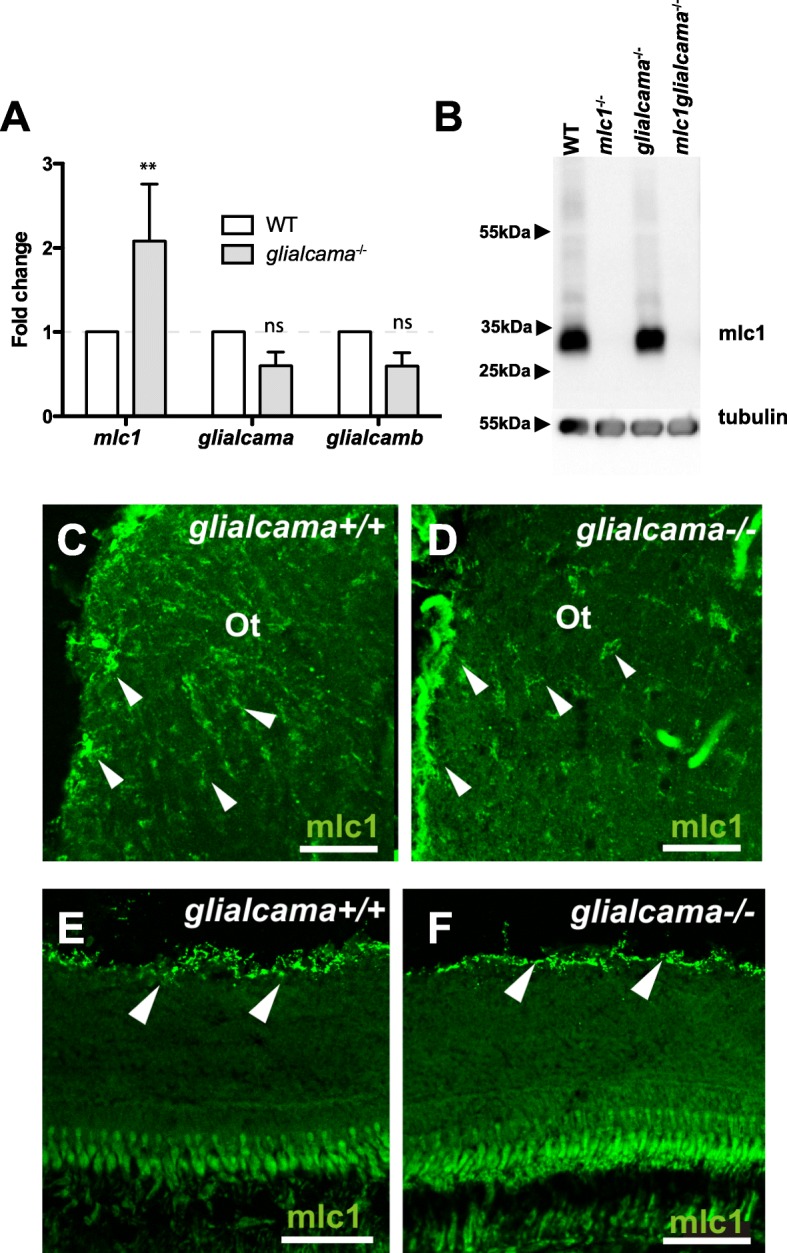
mlc1 expression and localization in *glialcama*^−/−^ zebrafish. **a** Quantitative real-time PCR to determine levels of *glialcama*, *glialcamb* and *mlc1* messenger RNA in the brain of *glialcama*^−/−^ zebrafish. Bars, relative expression levels compared with WT sibling; error bars, s.e. (*n* ≥ 3). **P < 0.01 (vs. wild-type, two-way ANOVA) **b** Comparison of mlc1 protein levels in brain of wild-type (WT), *mlc1*^−/−^, *glialcama*^−/−^ and *mlc1glialcama*^−/−^ zebrafish by Western blots of extracts from 5-months-old zebrafish. Western blot is representative of three independent experiments. Tubulin served as a loading control. **c**, **d** mlc1 expression (arrowheads) observed in the optic tract of both wild type (**c**) and *glialcama*^−/−^ (**d**) brains. **e**, **f** mlc1 expression is restricted to the inner limiting membrane of the retina (arrowheads) both in wild type (**e**) and *glialcama*^−/−^ (**f**). Scale bars: 50 μm (**c**, **d**); 60 μm (**e**, **f**)

We then analysed the expression of *glialcama*, *glialcamb* and *mlc1* in the brain of *glialcama*^−/−^ fish by quantitative real-time PCR (Fig. 4a). We observed that mRNA levels of *glialcama* and *glialcamb* were not changed. In contrast, the levels of *mlc1* messenger RNA in the brain were increased in *glialcama*^−/−^ zebrafish. It is interesting to point out that no changes in *Mlc1* messenger RNA levels were observed in *Glialcam*^−/−^ mice [15].

In mice, Mlc1 protein levels are strongly decreased or absent in *Glialcam*^−/−^ [15, 16]. We wondered whether the expression of mlc1 might be also changed in *glialcama*^−/−^ zebrafish. Unexpectedly, mlc1 protein levels were unchanged in *glialcama*^−/−^ zebrafish (Fig. 4b).

In mice, ablation of GlialCAM strongly reduced the labelling for Mlc1 and changed its localization in Bergmann glia and at astrocytic end-feet along blood vessels [15, 16]. We then studied mlc1 localization in the brain and retina of wild type and *glialcama*^−/−^ fish by immunofluorescence. We observed no detectable differences in mlc1 localization between wild type and *glialcama*^−/−^ fish either in the brain (Fig. 4c-d) or in the retina (Fig. 4e-f).

### Mlc1 is mislocalized in primary astrocytes from *Glialcam*^−/−^ mice

**Fig. 5:**
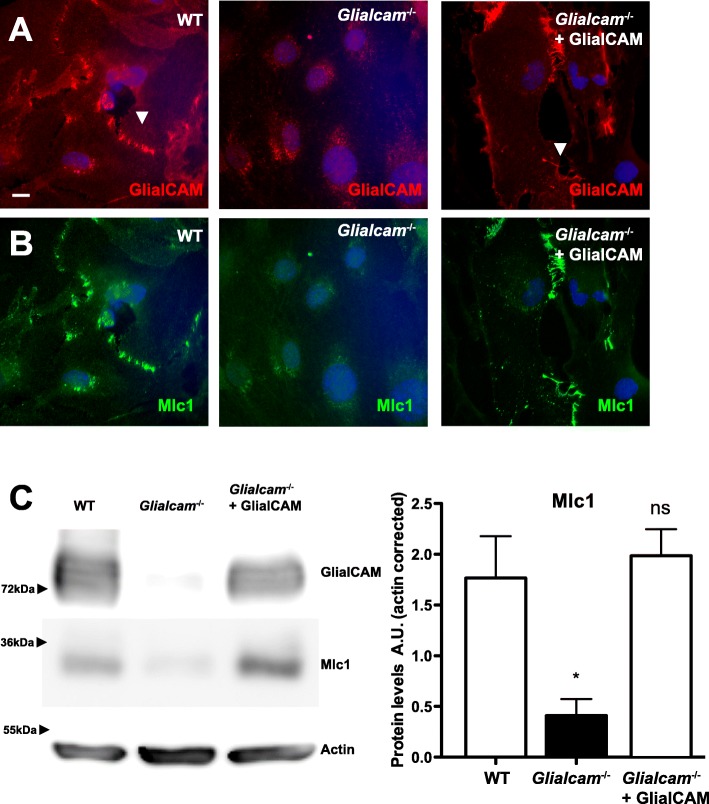
Mlc1 is mislocalized in primary *Glialcam*^−/−^ astrocytes. Localization of GlialCAM (**a**) and Mlc1 (**b**) in primary astrocytes from wild-type (WT, left), *Glialcam*^−/−^ (middle) and *Glialcam*^−/−^ complemented with adenoviruses expressing human GlialCAM (right). In WT and complemented astrocytes, GlialCAM and Mlc1 are located at cell-cell junctions (arrowheads). Scale bar: 10 μm. (**c**) GlialCAM and Mlc1 protein levels primary astrocytes from wild-type (WT, left), *Glialcam*^−/−^ (middle) and *Glialcam*^−/−^ complemented with an adenoviruss expressing human GlialCAM. Actin served as a loading control. Two other independent experiments gave similar results. Densitometric analysis (*n* = 3) indicates that Mlc1 levels were reduced in astrocytes from *Glialcam*^−/−^ mice and expression was recovered after expression of GlialCAM using adenoviruses. * *p* < 0.05 vs wild-type astrocytes

We wondered whether the differences observed in MLC1 expression and localization between *glialcama*^−/−^ zebrafish and *Glialcam*^−/−^ mice could be investigated in primary astrocyte cultures. In mouse primary astrocytes, lack of GlialCAM (Fig. 5a and c) caused a reduction of Mlc1 protein, as detected by Western blot (Fig. 5c) and a mislocalization of Mlc1, as it could not be detected in astrocyte junctions (Fig. 5b). Defects in Mlc1 protein expression and localization were rescued by expression of human GlialCAM using adenoviruses that expressed the protein (Fig. 5b and c). Thus, mouse primary *Glialcam*^−/−^ cultures recapitulated the Mlc1 expression defect and localization observed in vivo.

### Zebrafish mlc1 or human MLC1 overexpressed in primary astrocytes from *Glialcam*^−/−^ mice are located in cell junctions

**Fig. 6:**
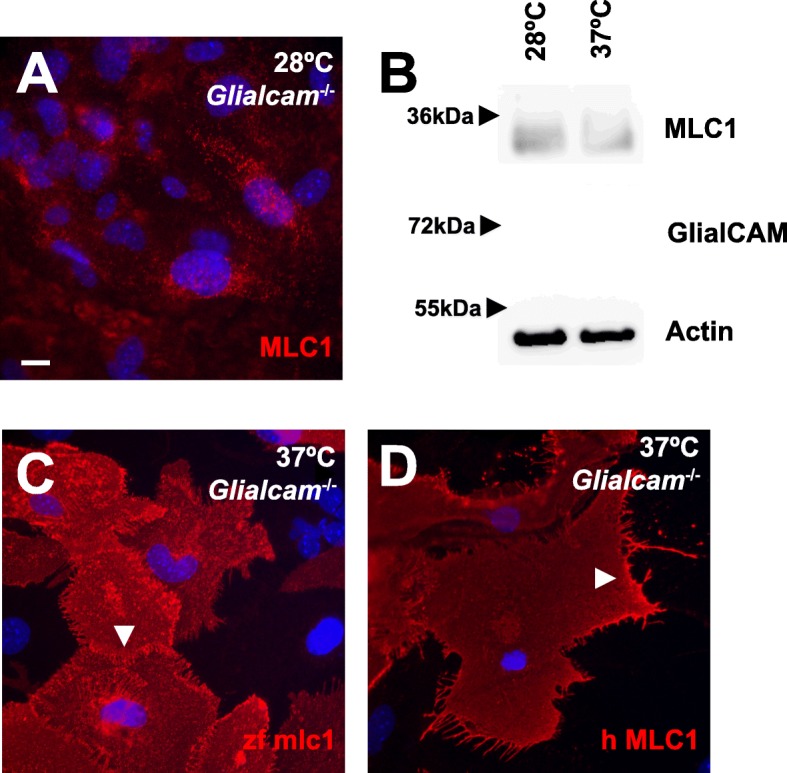
zfmlc1 and hMLC1 overexpressed in primary *Glialcam*^−/−^ astrocytes are located at cell-cell junctions. **a**, **b** Primary astrocytes isolated from *Glialcam*^−/−^ mice were incubated for 18 h at 28 °C. Then, MLC1 was detected by immunofluoresce (**a**) and protein levels were monitored by Western blot (**b**). Actin served as a loading control. Lack of signal using GlialCAM antibodies confirmed the lack of protein expression. **c**, **d** Overexpression using adenoviruses of mlc1 from zebrafish (zfmlc1, **c**) and human HA-tagged MLC1 (hMLC1, **d**) detected both MLC1 proteins at cell-cell junctions (arrowheads) in primary astrocytes isolated form *Glialcam*^−/−^ mice. We used antibodies detecting the zebrafish MLC1 or the HA epitope, which did not detect the endogenous Mlc1. Scale bar: 10 μm

We next investigated in *Glialcam*^−/−^ mouse primary astrocytes what reasons could explain the differences observed between mice and zebrafish regarding MLC1 protein levels and localization. As zebrafish are kept at 28 °C, which is a lower temperature than the temperature mice are kept (37 °C), we reasoned that stabilization of MLC1 by GlialCAM might not be necessary at lower temperatures. To test this hypothesis, we incubated mouse primary astrocytes at 28 °C overnight and assayed Mlc1 localization (Fig. 6a) and protein levels (Fig. 6b). However, no changes were observed at lower temperatures, suggesting that the stabilization of Mlc1 by GlialCAM is not temperature-dependent.

We then reasoned that the zebrafish mlc1 protein might not need glialcama for its stabilization at the plasma membrane, unlike their orthologs in mice and human. To test this hypothesis, we constructed an adenovirus expressing zebrafish mlc1 and infected *Glialcam*^−/−^ mouse primary astrocytes. Interestingly, zebrafish mlc1 was located at astrocyte junctions, suggesting that it may not need Glialcama for targeting to astrocyte junctions (Fig. 6c). To test that this was not the case for their human orthologous, we repeated the same experiment now with an adenovirus expressing human MLC1. Unexpectedly, human MLC1 overexpressed in *Glialcam*^−/−^ astrocytes was also located at astrocyte junctions (Fig. 6d).

## Discussion

In this work, we have obtained and characterized a *glialcama* knockout in zebrafish. The knockout displays megalencephaly and fluid accummulation, indicating that glialcama and not glialcamb, is the functional ortholog gene of GlialCAM in zebrafish. We do not know which could be the role of glialcamb in zebrafish. However, in vitro studies suggest the possibility that it may act as a negative regulator of MLC1 and ClC-2 [29, 32]. Taking into account that overexpression of MLC1 has been reported to be toxic in mice [33], there could be regulatory mechanisms inhibiting MLC1 function, such as interaction with glialcamb in zebrafish, although experimental evidence to support this hypothesis is lacking.

We also show that additional disruption of mlc1 in *glialcama* knockout zebrafish or in *Glialcam* knockout mice does not potentiate the vacuolating phenotype characteristic of MLC disease, indicating that loss-of-function mutations in these genes cause leukodystrophy through a common pathway. Previous [13] and recent [11] reports indicate that the phenotype of patients with mutations in *MLC1* is the same to those with recessive mutations in *GLIALCAM*. Thus, this genetic evidence in humans, together with biochemical studies in mice and zebrafish models of the disease and in vitro studies that indicated GlialCAM and MLC1 interaction, indicate that these proteins need to form a complex to carry out their physiological role. The situation is completely different for the ClC-2 protein. First, genetic evidence indicates that defects in *MLC1* or *CLCN2* lead to different diseases [34]. Second, the vacuolating phenotype of *Clcn2*^−/−^ mice increased after additional disruption of *Glialcam* [15]. Thus, we proposed that defects in ClC-2 might contribute partially to the MLC phenotype, but it is not the only reason to explain the phenotype of MLC patients.

The fact that the MLC1/GlialCAM complex is a functional unit is evident in the zebrafish knockout for *glialcama*, in which mlc1 protein is neither reduced nor mislocalized but yet it displays an MLC-like phenotype. In clear contrast, lack of *Mlc1* in mice or *mlc1* in zebrafish causes GlialCAM and glialcama mislocalization, respectively. Surprisingly, this localization defect could only be observed in primary cultured astrocytes from mouse after incubation with a depolarizing solution [29, 30]. Possibly, the mislocalization of GlialCAM when MLC1 is absent is a consequence of an unknown depolarization-dependent regulatory mechanism.

We speculate that mlc1 protein levels and localization in zebrafish are unaltered in the *glialcama*^−/−^, because in the zebrafish knockout there is an up-regulation of *mlc1* mRNA, which does not occur in the *Glialcam* knockout mice. In agreement with this hypothesis, in primary *Glialcam*^−/−^ astrocytes, where endogenous MLC1 is mislocalized, zebrafish or human MLC1 overexpressed are located at cell-cell junctions, suggesting that perhaps MLC1 overexpression compensates for lack of GlialCAM stabilizing effect.

Unlike in astrocytes, however, MLC1 overexpressed in cell lines without GlialCAM is never located at cell-cell junctions [25]. Possibly, in astrocytes, MLC1 may reach cell junctions not only by its interaction with GlialCAM, but also with the help of other proteins that may not be present in non-astrocyte cell lines.

## Conclusions

This work has provided new insights into the molecular interplay that exists between GlialCAM and MLC1, confirming that both proteins form a functional unit that is physiologically relevant. These results also indicate that in order to understand the molecular roles performed by the MLC1/GlialCAM complex, it is important to work at physiological protein levels, due to the fact that their overexpression may cause non-physiological effects [33].

## Supplementary information

**Additional file 1: Figure S1.** Co-immunoprecipitation of glialcama and mlc1 from brain zebrafish. Immunoprecipitation of glialcama from solubilized brain extracts (Sol) using an anti-glialcama polyclonal antibody coupled to Sepharose-A beads (IP +). Uncoupled beads were used as a negative control (IP -). The supernatant (SN) of both purifications is included. mlc1 was detected by Western blot. Another experiment gave similar results.

**Additional file 2: Figure S2.** T_2_ relaxation time measurement in the healthy and various brain regions of wild type, *mlc1* KO and *mlc1 glialcama* dKO mutant zebrafish. Region of interest (ROI) selected for T_2_ relaxation time measurements are shown in left images. ROI: (1) ventral telencephalon, (2) lesion in telencephalon, (3) lesion in mesencephalon, (4) ventricle.

**Additional file 3: Table S1.** Statistical comparison of wild type and mutant groups for percentage of area of Telencephalon with respect to whole brain (related to Fig. 2).

## Data Availability

All data generated or analysed during this study are included in this published article [and its Additional files].

## Abbreviations

MLC: Megalencephalic leukoencephalopathy with subcortical cysts
MRI: Magnetic resonance imaging
mRNA: messenger RNA
PCR: Polymerase chain reaction
VRAC: Volume regulated anion channel
