# Supplementary figures and images for: Comparison of zebrafish and mice knockouts for Megalencephalic Leukoencephalopathy proteins indicates that GlialCAM/MLC1 forms a functional unit
Source: Orphanet J Rare Dis. 2019 Nov 21;14:268. doi: 10.1186/s13023-019-1248-5 (PMC6873532; doi:10.1186/s13023-019-1248-5)

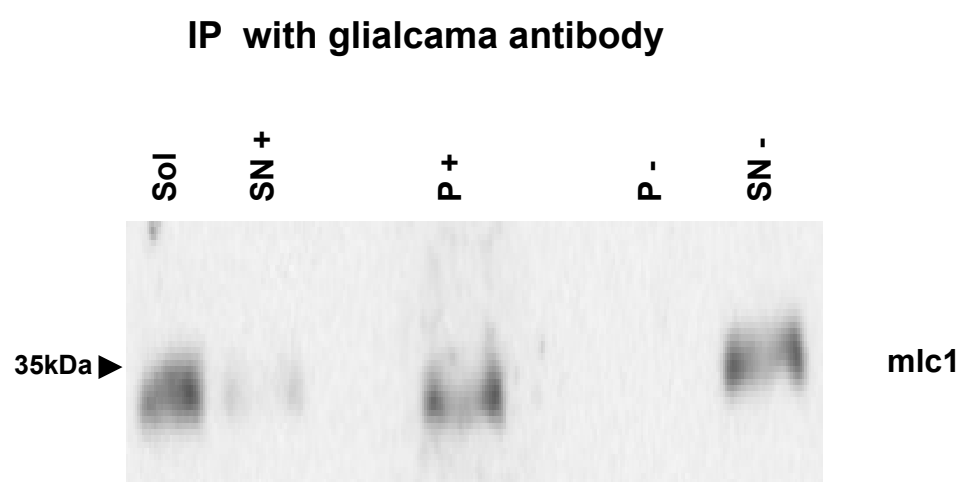

**Figure S1**

Supplement: Supplementary file 1 — Additional file 1: Figure S1. Co-immunoprecipitation of glialcama and mlc1 from brain zebrafish. Immunoprecipitation of glialcama from solubilized brain extracts (Sol) using an anti-glialcama polyclonal antibody coupled to Sepharose-A beads (IP +). Uncoupled beads were used as a negative control (IP -). The supernatant (SN) of both purifications is included. mlc1 was detected by Western blot. Another experiment gave similar results. [file 13023_2019_1248_MOESM1_ESM.pdf]
