# Supplementary material for: Comparison of zebrafish and mice knockouts for Megalencephalic Leukoencephalopathy proteins indicates that GlialCAM/MLC1 forms a functional unit
Source: Orphanet J Rare Dis. 2019 Nov 21;14:268. doi: 10.1186/s13023-019-1248-5 (PMC6873532; doi:10.1186/s13023-019-1248-5)
Supplement: Supplementary file 2 — Additional file 2: Figure S2. T2 relaxation time measurement in the healthy and various brain regions of wild type, mlc1 KO and mlc1 glialcama dKO mutant zebrafish. Region of interest (ROI) selected for T2 relaxation time measurements are shown in left images. ROI: (1) ventral telencephalon, (2) lesion in telencephalon, (3) lesion in mesencephalon, (4) ventricle. [file 13023_2019_1248_MOESM2_ESM.pdf]

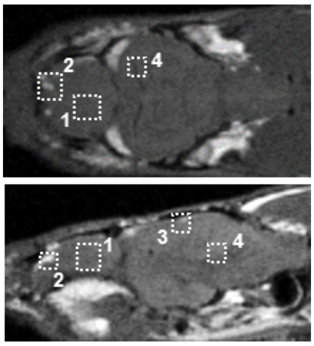

	Brain area	WT	*mlc1* ko	*mlc1* ko, *glialcama* ko
1	Ventral telencephalon	23.4±1.02	24.8±1.25	22.7±1.08
2	Lesions (in telencephalon)	-	30.9±1.68	29.2±1.12
3	Lesions (in mesencephalon)	-	26.6±1.57	29.4±1.57
4	Ventricle	34.6±3.16	36.1±2.74	33.1±1.90

Figure S2
