# Supplementary material for: Comparison of zebrafish and mice knockouts for Megalencephalic Leukoencephalopathy proteins indicates that GlialCAM/MLC1 forms a functional unit
Source: Orphanet J Rare Dis. 2019 Nov 21;14:268. doi: 10.1186/s13023-019-1248-5 (PMC6873532; doi:10.1186/s13023-019-1248-5)
Supplement: Supplementary file 3 — Additional file 3: Table S1. Statistical comparison of wild type and mutant groups for percentage of area of Telencephalon with respect to whole brain (related to Fig. 2). [file 13023_2019_1248_MOESM3_ESM.pdf]

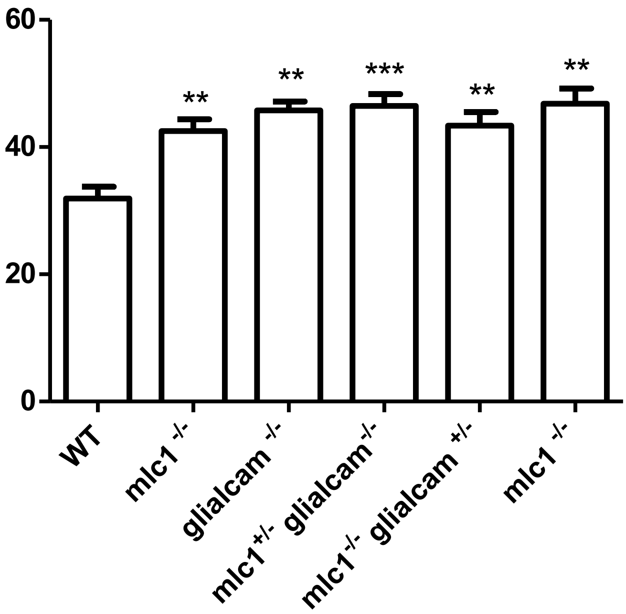

One-way analysis of variance			
P value	0.0013		
P value summary	**		
Are means signif. different? (P < 0.05)	Yes		
Number of groups	6		
F	9.053		
R squared	0.8045		
ANOVA Table	SS	df	MS
Treatment (between columns)	454.1	5	90.82
Residual (within columns)	110.4	11	10.03
Total	564.5	16	

Bonferroni's Multiple Comparison Test	Mean Diff.	t	Significant? P < 0.05?	Summary	95% CI of diff
WT vs $mlc1^{-/-}$	-10.60	4.099	Yes	*	-20.24 to -0.9578
WT vs $glialcam^{-/-}$	-13.87	5.362	Yes	**	-23.51 to -4.224
WT vs $mlc1^{+/-}$ $glialcam^{-/-}$	-14.53	5.620	Yes	**	-24.18 to -4.891
WT vs $mlc1^{-/-}$ $glialcam^{+/-}$	-11.47	4.434	Yes	*	-21.11 to -1.824
WT vs $mlc1^{-/-}$	-14.87	5.142	Yes	**	-25.65 to -4.086
$mlc1^{-/-}$ vs $glialcam^{-/-}$	-3.267	1.263	No	ns	-12.91 to 6.376
$mlc1^{-/-}$ vs $mlc1^{+/-}$ $glialcam^{-/-}$	-3.933	1.521	No	ns	-13.58 to 5.709
$mlc1^{-/-}$ vs $mlc1^{-/-}$ $glialcam^{+/-}$	-0.8667	0.3351	No	ns	-10.51 to 8.776
$mlc1^{-/-}$ vs $mlc1^{-/-}$	-4.267	1.476	No	ns	-15.05 to 6.514
$glialcam^{-/-}$ vs $mlc1^{+/-}$ $glialcam^{-/-}$	-0.6667	0.2578	No	ns	-10.31 to 8.976
$glialcam^{-/-}$ vs $mlc1^{-/-}$ $glialcam^{+/-}$	2.400	0.9280	No	ns	-7.242 to 12.04
$glialcam^{-/-}$ vs $mlc1^{-/-}$	-1.000	0.3458	No	ns	-11.78 to 9.780
$mlc1^{+/-}$ $glialcam^{-/-}$ vs $mlc1^{-/-}$ $glialcam^{+/-}$	3.067	1.186	No	ns	-6.576 to 12.71
$mlc1^{+/-}$ $glialcam^{-/-}$ vs $mlc1^{-/-}$	-0.3333	0.1153	No	ns	-11.11 to 10.45
$mlc1^{-/-}$ $glialcam^{+/-}$ vs $mlc1^{-/-}$	-3.400	1.176	No	ns	-14.18 to 7.380
